# Feasibility and Utility of Recumbent Ergometer-Based Cardiopulmonary Exercise Test in Phase 1 Cardiac Rehabilitation Following Cardiac Surgery: A Pilot Study

**DOI:** 10.3390/jcm15062429

**Published:** 2026-03-22

**Authors:** Yeon Mi Kim, Bo Ryun Kim, Ho Sung Son, Sung Bom Pyun, Jae Seung Jung, Hee Jung Kim

**Affiliations:** 1 Department of Physical Medicine and Rehabilitation, Korea University Anam Hospital, Seoul 02841, Republic of Korea; yeonmimi789@gmail.com (Y.M.K.);; 2 Department of Thoracic and Cardiovascular Surgery, Korea University Anam Hospital, Seoul 02841, Republic of Korea

**Keywords:** cardiac rehabilitation, cardiac surgery, exercise test, ergometry

## Abstract

**Background/Objectives:** Recent guidelines have emphasized the importance of early mobilization and rehabilitation of patients following cardiac surgery. However, studies on the optimal targets and prescription methods for phase I cardiac rehabilitation (CR) are lacking. This study aimed to evaluate the feasibility and utility of an early phase 1 submaximal cardiopulmonary exercise test (CPET) using a recumbent ergometer in patients who have undergone cardiac surgery. **Methods:** Twenty ambulatory patients who underwent cardiac surgery between December 2021 and February 2023 were referred to the CR department on the fifth postoperative day, and a CR program was initiated. The program was conducted five times a week, with hour-long sessions consisting of warm-up exercises, resistance training, aerobic exercises, and a cool-down period. A recumbent ergometer-based submaximal CPET was performed approximately nine days after the surgery, prior to discharge. Participants initiated the test at 0 W, and the workload was increased by 20 W after 2 min. During the test, researchers evaluated parameters including submaximal peak values of oxygen consumption (VO_2_), metabolic equivalents of task, respiratory exchange ratio (RER), blood pressure, heart rate (HR), and rating of perceived exertion (RPE). The grip strength test, 6 min walk test (6MWT), Korean Activity Scale/Index (KASI), EuroQol-5 dimension (EQ-5D), and short-form 36-item health survey (SF-36) values were also measured prior to discharge. **Results:** Twenty patients (75% male, average age 62.50 ± 1.99 years) underwent CPET at a median of 9.0 (8.0; 12.5) days postoperative. The average exercise duration of the CPET was 411.75 ± 168.25 s. During the test, their submaximal peak VO_2_ was 12.32 ± 0.75 mL/kg/min (corresponding to 46.65 ± 2.08% of VO_2_ max). The submaximal peak RER was 1.01 (0.98–1.12), and the submaximal peak RPE was 15.00 ± 0.51. Furthermore, the submaximal peak HR was 111.8 ± 3.76 beats/min (equivalent to 70.95 ± 2.09% of age-predicted maximal HR). After adjustment for age and sex, statistically significant positive correlations were observed between the submaximal peak VO_2_ and 6MWT, squat endurance test, KASI, EQ-5D, and the physical component summary (PCS) of the SF-36 questionnaire. The 6MWT, squat endurance test, KASI, and PCS of SF-36 showed a correlation coefficient (r) of 0.522 (*p* = 0.026), 0.628 (*p* = 0.005), 0.586 (*p* = 0.011), and 0.546 (*p* = 0.019), respectively. No significant cardiac events, such as ST elevation/depression or hemodynamic instability, were observed during the test. **Conclusions:** Our findings suggest that performing recumbent ergometer-based CPET during early phase 1 CR is safe and feasible. These results highlight the potential of recumbent ergometer-based CPET as a valuable tool for guiding the appropriate prescription of early CR programs following hospital discharge in patients undergoing cardiac surgery.

## 1. Introduction

Cardiovascular diseases (CVDs) represent a significant public health burden in South Korea. Over the past ten years, deaths attributable to CVD have risen by 25.2%, making it the second most common cause of mortality nationwide as of 2022. In addition, the increasing prevalence of hypertension and dyslipidemia among young adults is particularly concerning, as it may contribute to a future rise in CVD incidence. Each year, roughly 10,000 patients require intensive care unit admission following cardiovascular surgery, and this number showed an upward trend from 2010 to 2019. Surgical procedures associated with the highest death rates include those involving the aorta, coronary artery bypass grafting (CABG), and valve surgeries [1,2].

Cardiac rehabilitation (CR) is recognized for its benefits in enhancing exercise tolerance and quality of life and reducing the need for hospital readmissions and risk of death. Authoritative clinical practice guidelines from regions including Europe, America, and Korea have encouraged the integration of CR into the treatment plans of patients suffering from conditions such as congestive heart failure, valve-related diseases, coronary artery disease, and those recovering from heart surgeries [3,4,5,6].

CR is structured into three distinct stages. The initial stage, or phase 1, starts with the patient’s early movement activities in the ICU and ward to improve their daily living skills. This early stage typically involves supervised exercises within the hospital’s CR center, where the patient’s heart rate (HR) and electrocardiography (ECG) are closely monitored. Approximately 1–3 months post-surgery, following hospital discharge, patients return to the hospital for a symptom-limited cardiopulmonary exercise test (CPET). Depending on the test outcomes, patients identified as having moderate-to-high risk are recommended to participate in a supervised, center-based phase 2 CR program that includes thorough HR and ECG monitoring. Conversely, those deemed to be at low risk may be advised to undertake CR at home. The final stage, phase 3, encourages patients to commit to sustained CR exercises and risk factor management within their home and community settings, promoting indefinite, self-managed care and monitoring [7].

Symptom-limited treadmill-based CPET is widely recognized as a crucial component of the standard CR program. It evaluates a patient’s exercise capacity and prognosis by monitoring blood pressure, HR, ECG, and gas exchange as exercise intensity progressively increases. This detailed assessment helps in tailoring the right exercise prescription for CR [5]. Consequently, numerous clinical guidelines advocate for the administration of CPET prior to starting CR [8]. For instance, the Japanese guidelines suggest the implementation of a symptom-limited exercise stress test between 14 and 21 days following a myocardial infarction (MI) [9], and the ACC/AHA guidelines recommend that this test can be safely carried out between 3 and 6 weeks following MI [10].

However, these recommendations primarily apply to the subacute phase, and evidence supporting the safety and feasibility of treadmill-based CPET in the early postoperative period remains limited. Although one previous study reported the feasibility of a low-level treadmill-based CPET within four days after CABG, the sample size was very small [11]. Furthermore, nearly 20% of the patients were unable to undergo low-level CPET before discharge after MI because of medical instability, fear, or difficulty in walking on a treadmill [12]. In addition, treadmill-based CPET has been associated with a significantly higher incidence of overall cardiac events, premature ventricular contractions, angina, and ventricular tachycardia compared with cycle ergometer-based testing [13]. Cycle ergometer-based CPET, particularly using a recumbent cycle ergometer, may represent a safer alternative for patients in the early postoperative period. Unlike treadmill exercise, recumbent cycling is performed in a seated or semi-recumbent position, which minimizes balance demands and reduces the risk of falls. Moreover, as a non-weight-bearing modality, it requires less muscular effort, which makes it more suitable and potentially safer for patients recovering from cardiac surgery [14].

Recent studies have highlighted the advantages of initiating CR shortly after cardiac surgery, showing improvements in survival and shorter hospital stays without an increased risk of adverse events. Sezai et al. demonstrated that patients who began CR within a median of 1–3 days after open-heart surgery experienced lower in-hospital mortality, reduced length of stay, and decreased medical costs compared with those who did not receive early CR. Likewise, Pack et al. reported that it is safe to enroll patients in CR within two weeks after discharge, provided that individualized evaluation and tailored exercise prescriptions are conducted within a structured CR program [15,16].

Despite these findings, most previous studies and medical practices have traditionally scheduled CPET at approximately one month postoperatively [17]. Notably, guidelines regarding the safest timing for conducting CPET after cardiac surgery are lacking [8]. This one-month period after surgery is critical, as patients are at risk of falling into a state of physical inactivity, which has been linked to increased long-term mortality [18]. Implementing a safe and clinically informative submaximal CPET during the in-hospital phase 1 CR period may be crucial for the active application of early CR after cardiac surgery, as it can enhance both the safety and effectiveness of phase 1 CR and facilitate appropriate exercise prescription during the early transition to phase 2 CR. This study aimed to assess the safety, feasibility, and clinical utility of an early postoperative recumbent ergometer-based CPET performed within two weeks after open-heart surgery.

## 2. Methods

### 2.1. Study Objectives

The primary objective of this study was to evaluate the safety of early postoperative recumbent ergometer-based submaximal CPET performed within two weeks after open-heart surgery. Safety was assessed by monitoring the occurrence of major adverse cardiac events, hemodynamic instability, significant arrhythmias, abnormal blood pressure responses, or other clinically significant complications during testing. Secondary exploratory objectives included assessing the clinical utility of submaximal peak VO_2_ by examining its correlations with functional capacity measures and patient-reported outcomes.

### 2.2. Study Population

This prospective study was carried out at Korea University Anam Hospital and included patients who underwent open-heart surgery. The study protocol was approved by the Institutional Review Board of Korea University Anam Hospital (approval no: 2021AN0091). Between November 2021 and February 2023, patients referred for inpatient CR to the Department of Physical Medicine and Rehabilitation after open-heart surgery were screened for eligibility. Referrals were primarily made by the Department of Cardiothoracic Surgery, and eligibility was determined according to predefined inclusion and exclusion criteria. Written informed consent was obtained from all participants prior to enrollment. At Korea University Anam Hospital, approximately 140 patients per year are referred for inpatient CR following cardiac surgery. The present cohort was derived from a pilot study [19] requiring additional post-discharge follow-up and remote ECG monitoring, which limited enrollment due to patient preference.

Eligible participants were adults aged over 18 years with a left ventricular ejection fraction (LVEF) greater than 35% who were capable of performing an exercise test. Patients diagnosed with acute coronary syndrome, valvular heart disease, or aortic dissection who had undergone open-heart surgery and were able to ambulate independently were included.

Patients were excluded if they were classified as high-risk due to a history of cardiac arrest or the presence of an implantable cardioverter defibrillator, had unstable medical conditions, or exhibited cognitive impairments that interfered with understanding the exercise protocol. Individuals with musculoskeletal or neurological disorders affecting the lower extremities that limited exercise participation, as well as those unable to walk independently, were also excluded. In addition, patients with postoperative conditions considered unsafe for early CR—such as delayed sternal or surgical wound healing, significant postoperative anemia (e.g., hemoglobin < 10 g/dL), or other complications requiring prolonged medical stabilization—were excluded based on clinical judgment by the attending physician.

All patients who met the eligibility criteria and provided informed consent were consecutively enrolled.

### 2.3. Study Protocol

Starting on the fifth day after surgery, 20 patients participated in inpatient CR sessions. Sessions were held five times per week, lasted approximately one hour, and included warm-up exercises, resistance training, aerobic exercise, and cool-down activities. Before hospital discharge, which occurred around postoperative day nine, participants underwent a recumbent ergometer-based submaximal CPET along with the grip strength test, 6 min walk test (6MWT), Korean activity scale/index (KASI), EuroQol-5 dimension, and short-form 36-item health survey (SF-36).

**Figure 1:**
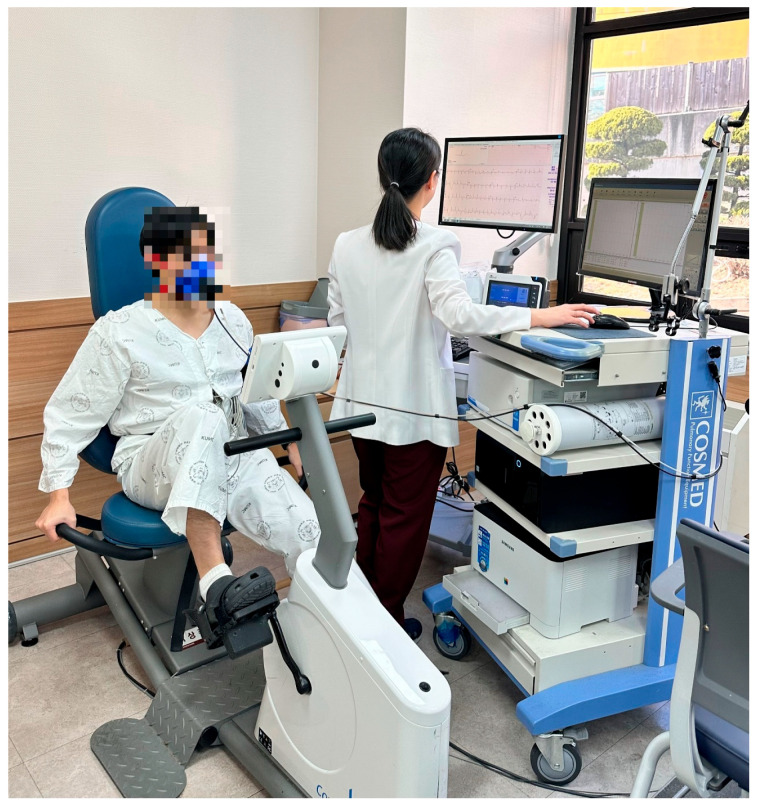
Submaximal cardiopulmonary exercise test with a recumbent ergometer.

Although treadmill-based CPET is commonly used because patients may discontinue cycling tests due to quadriceps fatigue and difficulty maintaining cadence [20,21], a recumbent ergometer was selected in this study to reduce physical strain on individuals in the early postoperative period (Figure 1). Participants were positioned on a recumbent ergometer (Corival Recumbent CPET 969900, Lode, Groningen, the Netherlands) and began pedaling at 60 rpm with an initial workload of 0 W. The workload was increased by 20 W every two minutes. Testing was terminated when absolute indications for discontinuation or predefined termination criteria were met (Table 1). Throughout the test, HR, oxygen consumption (VO_2_), metabolic equivalents of task (MET), respiratory exchange ratio (RER), rating of perceived exertion (RPE), blood pressure (BP), dyspnea and angina scale, total exercise duration, and maximal work capacity (Wmax) were recorded. Wmax was defined as the highest workload that could be sustained for at least 30 s.

“Submaximal peak VO_2_” was defined as the highest VO_2_ value achieved during the test prior to meeting termination criteria. Because the protocol was submaximal and included predefined heart rate and perceived exertion limits, these values do not represent true maximal oxygen uptake (VO_2_ max).

The grip strength test, a widely used indicator of muscle strength and an important predictor of cardiovascular outcomes, hospitalization duration, functional status, and perioperative complication [22,23,24], was measured using a digital dynamometer (JAMAR PLUS+ Digital Hand Dynamometer; Sammons Preston Rolyan, Bolingbrook, IL, USA). Participants performed two maximal efforts with each hand, and the highest value (kg) was recorded. Since most participants were right-handed, only right-hand grip strength was analyzed.

The 6MWT was conducted to assess functional capacity and daily activity performance [25]. Participants walked along a 30 m corridor for six minutes, aiming to cover the greatest possible distance while maintaining a perceived exertion level of 3 to 4 (moderate to somewhat strong) on the Borg CR scale 10 [26]. Rest was allowed if needed, and the test was discontinued in cases of dyspnea or chest pain that interfered with daily activities. Participants were informed of the elapsed time at one-minute intervals without encouragement. Heart rate, blood pressure, and oxygen saturation were measured before and after the test, and total walking distance was recorded.

The KASI [27] is a Korean adaptation of the Duke Activity Status Index (DASI). The DASI is a 12-item questionnaire designed to evaluate functional capacity and quality of life and has been shown to correlate with peak oxygen uptake and major adverse cardiac events [28]. Participants completed the KASI independently, and total scores were calculated based on their responses.

The EuroQol-5 dimension (EQ-5D) questionnaire assesses health-related quality of life in five dimensions: mobility, self-care, usual activities, pain/discomfort, and anxiety/depression [29]. Each dimension has three response levels (no problems, moderate problems, severe problems). Responses were converted into index scores using Korean population value sets, ranging from −0.171 to 1. A score of 1 indicates perfect health, 0 corresponds to death, and negative values represent health states worse than death [30,31].

The SF-36 questionnaire was used to assess overall quality of life across eight domains: physical functioning, role limitations due to physical problems, bodily pain, general health, vitality, social functioning, role limitations due to emotional problems, and mental health. These domains produce scores ranging from 0 (worst health) to 100 (best health). The results can be summarized into the Physical Component Summary (PCS) and Mental Component Summary (MCS), both standardized to a mean of 50 with a standard deviation of 10 in the general population [32]. The Korean version of the SF-36 was used to ensure cultural relevance [33].

### 2.4. Statistical Analysis

SPSS version 26 (IBM Corp., Armonk, NY, USA) was used for the statistical analysis. Analyses involving echocardiographic parameters and NT-proBNP were conducted using available data only (complete-case analysis), and no imputation was performed for missing values. Normality of continuous variables was assessed using the Shapiro–Wilk test. As several variables were not normally distributed, nonparametric methods were applied. Spearman’s partial correlation analyses adjusting for age and sex were performed to evaluate the associations between the 6MWT, self-reported questionnaire outcomes (KASI, EQ-5D, SF-36), and submaximal peak VO_2_ obtained during the exercise test. Confidence intervals for correlation coefficients were calculated using Fisher’s z transformation. Statistical significance was set at a *p*-value < 0.05.

## 3. Results

### 3.1. Baseline Characteristics

Twenty patients were recruited between November 2021 and April 2023. All participants successfully completed the CPET protocol without premature termination, and no major adverse cardiac events occurred during testing. The baseline characteristics of the study participants are presented in Table 2. The average age of the patients was 62.50 years (15 males, 5 females). Atrial fibrillation was present in 25% of the participants at baseline. The median time to initiate inpatient CR was 5 days, while the median time to undergo CPET was 9 days postoperative. The most common reasons for surgery were infective endocarditis and valve diseases, each accounting for 25% of the cases, followed by stable angina (20%) and MI (15%). Ten patients underwent valve surgery, while eight patients underwent CABG surgery. The remaining two patients received total arch replacement and coronary artery fistulectomy.

### 3.2. Assessments of Functional Capacity

Table 3 displays the outcomes of functional capacity measured at an average of nine days after surgery. The average grip strength of the right hand was 29.93 kg (±1.71). The 6MWT was 350.15 m (±18.85). The average scores for KASI and SF-36 were 10.26 (±0.77) and 106.48 (±3.13), respectively. The median value of EQ-5D was 0.72 (range: 0.68–0.72) (Table 3).

### 3.3. Results of Recumbent Ergometer-Based Submaximal CPET

During the test, the average duration of the CPET was 411.75 ± 168.25 s. The average submaximal peak VO_2_ was 12.32 ± 0.75 mL/kg/min, corresponding to 46.65 ± 2.08% of VO_2_ max. The average values for submaximal peak MET and Wmax were 3.52 ± 0.22 and 63.00 ± 7.00 W, respectively. The submaximal peak RER was 1.01 (range: 0.98–1.12), and the peak RPE was 15.00 ± 0.51. Additionally, the submaximal peak HR was 111.8 ± 3.76 beats/min, equivalent to 70.95 ± 2.09% of the age-predicted maximal HR (Table 4).

After adjusting for age and sex, Spearman’s correlation analysis demonstrated a statistically significant positive correlations between the submaximal peak VO_2_ and the 6MWT (r = 0.52, *p* = 0.026; 95% CI 0.057–0.789), KASI (r = 0.59, *p* = 0.011; 95% CI 0.147–0.821), and PCS of the SF-36 questionnaire (r = 0.546, *p* = 0.019; 95% CI 0.090–0.801). No significant correlations were found between the submaximal peak VO_2_ and other measurements (Table 5).

Throughout the test, no patients experienced significant cardiac events, such as ST-segment elevation or depression, or anginal chest pain. The reasons for test termination were shortness of breath in 10 patients and leg discomfort in nine patients. One patient stopped the test because of a combination of shortness of breath and leg discomfort, and another patient stopped because of leg discomfort accompanied by a decrease in BP. In the patient who exhibited a drop in BP, the resting BP was 129/93 mmHg. During stage 3 of the test, the BP decreased from 144/86 mmHg at stage 2 to 116/89 mmHg, and the test was terminated according to the predefined termination criteria (Table 1).

### 3.4. Discussion

Our findings further support the idea that initiating submaximal exercise testing within 2 weeks (an average of 9 days) after cardiac surgery can be safely performed. Hamm et al. suggested that patients who underwent exercise testing 15–28 days after acute MI had a significantly higher rate of cardiac arrest compared with those tested within 14 days after acute MI [34]. Miller et al. found that performing an exercise test soon after MI is beneficial to promote patients’ self-confidence, guide post-discharge exercise prescriptions, and predict post-hospital prognosis. However, they emphasized that such tests should be conducted in a well-controlled research environment due to safety concerns [35]. A previous study on early symptom-limited exercise stress test after PTCA or CABG found that the main reasons for early termination of the test were chest pain, ST changes, hemodynamic instability, dyspnea, and musculoskeletal pain [36]. In our study using a recumbent ergometer-based submaximal CPET, none of the patients experienced ST changes, hemodynamic instability, or chest pain.

In the present study, no major adverse cardiac events, including ST-segment changes, hemodynamic instability, or chest pain, were observed during CPET. While this absence of adverse events is reassuring, the findings should be interpreted cautiously. The study cohort comprised a small number of relatively low-risk patients who were ambulatory, medically stable, and had preserved left ventricular function (LVEF > 35%), with higher-risk patients excluded by design. Moreover, the termination criteria applied in this study were relatively conservative, which may have contributed to the favorable safety profile observed. Consequently, these results cannot be readily extrapolated to higher-risk postoperative populations, and the external validity of the safety findings is limited. These methodological considerations should be carefully taken into account when interpreting the safety of early postoperative CPET.

A previous study found that the results of a submaximal exercise test using a treadmill, performed 5.0 ± 2.8 days after admission for unstable angina, were useful in predicting outcomes during the first year after hospital discharge [37]. However, few studies have examined submaximal testing early after cardiac surgery. Although VO_2_ max obtained from a maximal treadmill test is 6–17% higher than that obtained from a maximal cycle ergometer test due to the engagement of larger muscle groups during treadmill exercise, a linear increase in work rate on both treadmill and cycle ergometer can effectively demonstrate oxygen uptake patterns during CPET [38,39]. We chose the recumbent ergometer for the submaximal exercise test because it carries a lower risk of falls and allows for easier acquisition of ECG data with fewer artifacts.

Despite these limitations, the consistency of our findings with previously reported results serves to support the validity of our approach. Takeyama et al. [40] studied 28 patients who underwent CABG and divided them into training and control groups, with exercise training initiated one week after surgery. All patients underwent CPET using a cycle ergometer. Peak VO_2_ measured during CPET was 13.1 ± 1.7 mL/kg/min in the training group (mean age 58.8 ± 6.3 years) and 13.7 ± 2.5 mL/kg/min in the control group (mean age 61.7 ± 8.7 years). In the present study, peak VO_2_ measured at a median of 9 days after surgery was 12.32 ± 0.75 mL/kg/min. Considering the relatively higher mean age of our study population (62.5 years) and the greater proportion of female patients, the similarity in peak VO_2_ values suggests a strong correlation with previous findings, supporting the validity of early submaximal CPET after cardiac surgery.

However, some methodological differences exist between the study by Takeyama et al. and our study. First, unlike our study, which utilized a recumbent ergometer, their study used an upright cycle ergometer. In our cohort of patients who had recently undergone open-heart surgery, a recumbent ergometer was used to minimize the risk of falls and enhance safety during early postoperative testing. Previous studies have demonstrated that upright cycle ergometers generally yield higher peak VO_2_ values than recumbent ergometers at submaximal workloads [41], which may partially account for the slightly higher oxygen consumption values observed by Takeyama et al. Additionally, although Takeyama et al. conducted CPET early (one week after surgery), they did not examine the correlations between peak VO_2_ and other functional measures. As a result, the reliability of CPET-derived aerobic capacity in the early postoperative phase could not be fully assessed in their study. To address this limitation, we evaluated correlations between CPET parameters and multiple functional and patient-reported outcomes, including the 6MWT, KASI, and the PCS domain of SF-36, thereby providing further evidence supporting the validity of early submaximal CPET after cardiac surgery.

The submaximal peak VO_2_ in our study also showed statistically significant correlations with the 6MWT, KASI, and PCS of the SF-36 questionnaires. Pauletti et al. demonstrated that 6MWT performed on postoperative day 5 after cardiac surgery can predict clinical outcomes and patient recovery [42], and it has been widely used to evaluate aerobic capacity before and after cardiac rehabilitation [43]. Submaximal exercise testing offers additional advantages beyond functional walking tests, as it allows assessment of physiological parameters such as oxygen consumption, HR, and METs. Consistent with prior studies demonstrating moderate to high correlations between the physical function domain of the SF-36 and 6MWT performance [44], our findings suggest that submaximal peak VO_2_ reflects aspects of postoperative physical function and health-related quality of life. As the protocol was intentionally submaximal, the submaximal peak VO_2_ values should be interpreted as modality-specific reference points rather than absolute physiological thresholds. In the early postoperative setting, their primary utility lies in providing an individualized basis for conservative, symptom-limited exercise prescription. However, the small sample size resulted in relatively wide confidence intervals, and the study may have been underpowered to detect modest associations. In addition, the cohort included heterogeneous surgical populations, and subgroup analyses were not performed due to limited numbers within each category. As an exploratory pilot study, no formal a priori sample size calculation or adjustment for multiple comparisons was conducted; therefore, the findings should be interpreted as hypothesis-generating rather than confirmatory.

Importantly, the present study was designed as a preliminary feasibility investigation conducted during a very early postoperative period (within 14 days after cardiac surgery), a time window in which exercise testing is generally considered to carry increased risk. To prioritize patient safety in this vulnerable phase, we deliberately employed a recumbent ergometer-based submaximal CPET protocol. This approach differs from most previous studies, which have focused on later postoperative testing or treadmill- or upright cycle-based protocols. In addition, by demonstrating statistically significant correlations between submaximal peak VO_2_ and established functional and patient-reported outcome measures, our study extends existing knowledge by providing early evidence supporting both the feasibility and clinical relevance of recumbent submaximal CPET in the immediate postoperative setting. Taken together, these findings should be interpreted as preliminary evidence that provides a foundation for future larger-scale, adequately powered studies incorporating broader risk profiles and evaluating the clinical impact of early recumbent CPET-guided exercise prescription on postoperative recovery and long-term outcomes.

### 3.5. Limitations

This study had several important limitations. First, the small sample size, predominance of male participants, absence of a control group and randomization, and heterogeneity of underlying cardiac diagnoses may have limited the statistical power of the analyses and compromised internal validity, thereby restricting the generalizability of the findings. In addition, the correlation analyses were based on a limited number of participants, resulting in relatively wide confidence intervals for the correlation coefficients. This indicates uncertainty in the precision of these estimates and suggests that the observed associations should be interpreted cautiously as exploratory findings rather than definitive conclusions. Second, this study did not maintain a prospective screening log of all patients referred to for inpatient CR during the study period. Therefore, the proportion of eligible patients relative to the total referred population could not be determined, which may limit the interpretation of feasibility. However, all patients who met the predefined inclusion and exclusion criteria and provided informed consent were consecutively enrolled, which helps to minimize selection bias. Third, the use of a recumbent ergometer rather than a treadmill may have resulted in an underestimation of peak oxygen consumption [20]; however, this approach was deliberately chosen to minimize cardiovascular and musculoskeletal stress in patients during the early postoperative period following cardiac surgery. Future studies employing randomized controlled designs, larger cohorts, and more homogeneous diagnostic populations are warranted to more clearly define the role of early recumbent CPET in guiding individualized CR.

## 4. Conclusions

The findings of this study suggest that CPET using a recumbent ergometer appears to be safe and feasible in carefully selected, low-risk patients within two weeks after cardiac surgery when performed in a controlled inpatient setting. Early initiation of CPET was not associated with an increased incidence of adverse events in this cohort, indicating potential safety in the early postoperative period. In addition, peak VO_2_ values obtained during submaximal CPET may offer potentially useful physiological information relevant to exercise intensity setting in the early rehabilitation phase. While these results should be interpreted cautiously, the present study is exploratory in nature and provides preliminary evidence to support further investigation of early recumbent CPET as a feasible assessment tool for informing individualized exercise intensity during phase I or early phase II CR in larger, adequately powered randomized studies.

## Figures and Tables

**Table 1:** Termination criteria for recumbent ergometer-based submaximal exercise test.

Absolute Indication for Stop Test	Pre-Established Termination Criteria
-ST-segment elevation > 1 mm without abnormal Q waves in ECG channels excluding V1 and aVR -Decrease in SBP > 10 mmHg or a drop below resting SBP -Severe or higher-grade angina (angina scale Grade 3–4) -Able to ambulate without physical assistance -Exacerbation of neurological symptoms (ex. dizziness and ataxia) -Cyanosis or pallor -Persistent ventricular tachycardia -Patient’s desire to discontinue the test	-HR > 120 beats per minute. -Reaching 70% of the maximum predicted heart rate based on the patient’s age. -Achieving a pre-determined MET level (typically 5–7). -Uncontrolled medical condition -RPE (Rating of Perceived Exertion) exceeds 15 on the Borg 6–20 grade scale.

**Table 2:** Baseline characteristics of the subjects (*N* = 20).

Variables	Values
Age (years)	62.50 ± 1.99
Sex, males/females	15 (75)/5 (25)
Height (cm)	166.14 ± 1.99
Weight (kg)	66.28 ± 1.82
Body mass index (kg/m^2^)	23.97 ± 0.43
Normal weight (18.5–22.9)	5 (25)
Overweight (23.0–24.9)	8 (40)
Obese (≥25.0)	7 (35)
Atrial fibrillation	5 (25)
NT-pro BNP (pg/mL, *N* = 15)	597 (129; 1555)
E/e’ ratio (*N* = 15)	10.9 (9.3; 14.6)
Left Atrial volume index (mL/m^2^, *N* = 16)	55.1 (33.1; 68.9)
Time to start CR (days)	5.0 (5.0; 6.0)
Time to CPET (days)	9.0 (8.0; 12.5)
Surgery type (%)	
Valve surgery	10 (50)
CABG	8 (40)
Total arch replacement	1 (5)
Coronary artery fistulectomy	1 (5)
Cause of surgery (%)	
Infective endocarditis	5 (25)
Valve disease	5 (25)
Stable angina	4 (20)
Myocardial infarction	3 (15)
Unstable angina	1 (5)
Acute Aortic Dissection (DeBakey type 1)	1 (5)
Coronary arteriovenous fistula	1 (5)

Values represent mean ± standard deviation, median (interquartile range) or number (%) of cases Body mass index categories were defined according to the World Health Organization Asia-Pacific guidelines. NT-pro BNP, N-Terminal Brain Natriuretic Peptides; CR, cardiac rehabilitation; CPET, cardiopulmonary exercise test; CABG, coronary artery bypass grafting.

**Table 3:** Functional capacity measurement results (*N* = 20).

Variables	Values
Grip strength test (kg)	29.93 ± 1.71
6MWT (m)	350.15 ± 18.85
KASI	10.26 ± 0.77
EQ-5D	0.72 (0.68; 0.72)
SF-36	
Total	106.48 ± 3.13
PCS	47.14 ± 1.57
MCS	59.34 ± 2.18

Values represent mean ± standard deviation or median (interquartile range). 6MWT, 6 min walk test; KASI, Korean activity scale/index; EQ-5D, EuroQol-5 dimension; SF-36, short-form 36-item health survey; PCS, physical component summary; MCS, mental component summary.

**Table 4:** Results of a recumbent ergometer-based submaximal CPET (*N* = 20).

	Values
CPET duration (second)	411.75 ± 168.25
Submaximal peak VO_2_ (mL/kg/min)	12.32 ± 0.75
Percentage of VO_2_ max (%)	46.65 ± 2.08
Submaximal peak MET	3.52 ± 0.22
Submaximal peak RER	1.01 (0.98; 1.12)
Wmax (Watt)	63.00 ± 7.00
Submaximal peak RPE	15.00 ± 0.51
Submaximal peak HR (beats/min)	111.8 ± 3.76
Percentage of age-predicted maximal HR (%)	70.95 ± 2.09
Submaximal peak systolic BP (mmHg)	162.60 ± 4.47

Values represent mean ± standard deviation or median (interquartile range). CPET, cardiopulmonary exercise test; VO_2_ max, maximal oxygen consumption; MET, metabolic equivalents of task; RER, respiratory exchange ratio; Wmax, maximal work capacity; RPE, rating of perceived exertion; HR, heart rate; BP, blood pressure.

**Table 5:** Correlation between submaximal peak VO_2_ and other measures adjusting age and sex (*N* = 20).

Variables	Partial Correlation Coefficient	95% CI	*p*-Value
6MWT (m)	0.522 *	0.057–0.789	0.026
Grip strength test	0.065	−0.416–0.514	0.799
KASI	0.586 *	0.147–0.821	0.011
EQ-5D	−0.111	−0.547–0.378	0.662
SF-36			
Total	0.438	−0.049–0.746	0.069
PCS	0.546 *	0.090–0.801	0.019
MCS	0.194	−0.305–0.602	0.440

*, *p* < 0.05, statistically significant correlations. CI, confidence interval, 6MWT, 6 min walk test; KASI, Korean activity scale/index; EQ-5D, EuroQol-5 dimension; SF-36, short-form 36-item health survey; PCS, physical component summary; MCS, mental component summary.

## Data Availability

The original contributions presented in this study are included in the article. Further inquiries can be directed to the corresponding author.

## Abbreviations

CVD	Cardiovascular disease
CABG	coronary artery bypass grafting
CR	cardiac rehabilitation
HR	heart rate
ECG	electrocardiogram
CPET	cardiopulmonary exercise test
CBCR	center-based cardiac rehabilitation
HBCR	home-based cardiac rehabilitation
6MWT	6 min walk test
EQ-5D	EuroQol-5 dimension
SF-36	short-form 36-item health survey
PCS	Physical component summary
MCS	Mental component summary
KASI	Korean Activity Scale/Index
MET	metabolic equivalent of task
Wmax	maximal work capacity
RPE	rate of perceived exertion

## References

[1] Nam S.W., Song I.A., Oh T.K. (2023). Trends in Cardiovascular Surgery in South Korea: A Nationwide Cohort Study from 2010 to 2019. J. Cardiothorac. Vasc. Anesth..

[2] Kang H.J., Wang J., Cho E.J., Kim H.L., Lee C.J., Park J.H., Kang J., Suh J., Choi K.H., Lee S.Y. (2025). Addressing Cardiovascular Diseases Challenges in South Korea: Strategies to Improve Outcomes. Korean Circ. J..

[3] Heidenreich P.A., Fonarow G.C., Breathett K., Jurgens C.Y., Pisani B.A., Pozehl B.J., Spertus J.A., Taylor K.G., Thibodeau J.T., Yancy C.W. (2020). 2020 ACC/AHA clinical performance and quality measures for adults with heart failure: A report of the American College of Cardiology/American Heart Association Task Force on Performance Measures. Circ. Cardiovasc. Qual. Outcomes.

[4] Visseren F.L.J., Mach F., Smulders Y.M., Carballo D., Koskinas K.C., Bäck M., Benetos A., Biffi A., Boavida J.-M., Capodanno D. (2021). 2021 ESC Guidelines on cardiovascular disease prevention in clinical practice: Developed by the Task Force for cardiovascular disease prevention in clinical practice with representatives of the European Society of Cardiology and 12 medical societies With the special contribution of the European Association of Preventive Cardiology (EAPC). Eur. Heart J..

[5] Kim C., Sung J., Lee J.H., Kim W.S., Lee G.J., Jee S., Jung I.Y., Rah U.W., Kim B.O., Choi K.H. (2019). Clinical Practice Guideline for Cardiac Rehabilitation in Korea. Ann. Rehabil. Med..

[6] Varma N., Marrouche N.F., Aguinaga L., Albert C.M., Arbelo E., Choi J.-I., Chung M.K., Conte G., Dagher L., Epstein L.M. (2021). HRS/EHRA/APHRS/LAHRS/ACC/AHA worldwide practice update for telehealth and arrhythmia monitoring during and after a pandemic: Developed in partnership with and endorsed by the American College of Cardiology (ACC), the American Heart Association (AHA), the Asia Pacific Heart Rhythm Society (APHRS), the European Heart Rhythm Association (EHRA), the Heart Rhythm Society (HRS), and the Latin American Heart Rhythm Society (LAHRS). EP Eur..

[7] Ambrosetti M., Abreu A., Corrà U., Davos C.H., Hansen D., Frederix I., Iliou M.C., Pedretti R.F.E., Schmid J.P., Vigorito C. (2021). Secondary prevention through comprehensive cardiovascular rehabilitation: From knowledge to implementation. 2020 update. A position paper from the Secondary Prevention and Rehabilitation Section of the European Association of Preventive Cardiology. Eur. J. Prev. Cardiol..

[8] Price K.J., Gordon B.A., Bird S.R., Benson A.C. (2016). A review of guidelines for cardiac rehabilitation exercise programmes: Is there an international consensus?. Eur. J. Prev. Cardiol..

[9] Nohara R., Adachi H., Goto Y., Hasegawa E., Ishihara S., Itoh H., Kimura Y., Maehara K., Makita S., Matsuo H. (2014). Guidelines for Rehabilitation in Patients with Cardiovascular Disease (JCS 2012)—Digest Version. Circ. J..

[10] Gibbons R.J., Beasley J.W., Duvernoy W.F.C., Mark D.B., McCallister B.D., Winters W.L., Ritchie J.L., Cheitlin M.D., Eagle K.A., Gardner T.J. (1997). ACC/AHA guidelines for exercise testing: Executive summary—A report of the American College of Cardiology American Heart Association task force on practice guidelines (Committee on exercise testing). Circulation.

[11] Christopherson D.J., Shively M., Sivarajan E.S. (1984). Low-level exercise testing before and after coronary artery bypass surgery. Int. J. Nurs. Stud..

[12] Krone R.J., Gillespie J.A., Weld F.M., Miller J.P., Moss A.J. (1985). Low-level exercise testing after myocardial infarction: Usefulness in enhancing clinical risk stratification. Circulation.

[13] Ren C., Zhu J.X., Shen T., Song Y.X., Tao L.Y., Xu S.L., Zhao W., Gao W. (2022). Comparison Between Treadmill and Bicycle Ergometer Exercises in Terms of Safety of Cardiopulmonary Exercise Testing in Patients with Coronary Heart Disease. Front. Cardiovasc. Med..

[14] Le J.N., Zhou R., Tao R., Dharmavaram N., Dhingra R., Runo J., Forfia P., Raza F. (2023). Recumbent Ergometer vs Treadmill Cardiopulmonary Exercise Test in HFpEF: Implications for Chronotropic Response and Exercise Capacity. J. Card. Fail..

[15] Sezai A., Shimokawa T., Kanaoka K., Fukuma N., Sekino H., Shiraishi H., Sumita Y., Nakai M., Iwanaga Y., Furukawa Y. (2022). Efficacy of Early Cardiac Rehabilitation After Cardiac Surgery—Verification Using Japanese Diagnosis Procedure Combination Data. Circ. Rep..

[16] Pack Q.R., Dudycha K.J., Roschen K.P., Thomas R.J., Squires R.W. (2015). Safety of Early Enrollment into Outpatient Cardiac Rehabilitation After Open Heart Surgery. Am. J. Cardiol..

[17] Nilsson H., Nylander E., Borg S., Tamás É., Hedman K. (2019). Cardiopulmonary exercise testing for evaluation of a randomized exercise training intervention following aortic valve replacement. Clin. Physiol. Funct. Imaging.

[18] Kim S.H., Cha S., Kang S., Han K., Paik N.J., Kim W.S. (2021). High prevalence of physical inactivity after heart valve surgery and its association with long-term mortality: A nationwide cohort study. Eur. J. Prev. Cardiol..

[19] Kim Y.M., Kim B.R., Pyun S.B., Jung J.S., Kim H.J., Son H.S. (2025). Feasibility and Safety of Early Cardiac Rehabilitation Using Remote Electrocardiogram Monitoring in Patients with Cardiac Surgery: A Pilot Study. J. Clin. Med..

[20] Balady G.J., Arena R., Sietsema K., Myers J., Coke L., Fletcher G.F., Forman D., Franklin B., Guazzi M., Gulati M. (2010). Clinician’s Guide to cardiopulmonary exercise testing in adults: A scientific statement from the American Heart Association. Circulation.

[21] Lockwood P.A., Yoder J.E., Deuster P.A. (1997). Comparison and cross-validation of cycle ergometry estimates of VO2max. Med. Sci. Sports Exerc..

[22] Bohannon R.W. (2008). Hand-grip dynamometry predicts future outcomes in aging adults. J. Geriatr. Phys. Ther..

[23] Rantanen T., Guralnik J.M., Foley D., Masaki K., Leveille S., Curb J.D., White L. (1999). Midlife hand grip strength as a predictor of old age disability. Jama.

[24] Fu L.Y., Zhang Y.Y., Shao B.H., Liu X.J., Yuan B., Wang Z.Q., Chen T.A., Liu Z.G., Liu X.C., Guo Q. (2019). Perioperative poor grip strength recovery is associated with 30-day complication rate after cardiac surgery discharge in middle-aged and older adults-a prospective observational study. Bmc Cardiovasc. Disord..

[25] Enright P.L. (2003). The six-minute walk test. Respir. Care.

[26] Borg G.A.V. (1982). Psychophysical Bases of Perceived Exertion. Med. Sci. Sports Exerc..

[27] Sung J., On Y.K., Kim H.S., Chae I.H., Sohn D.W., Oh B.H., Lee M.M., Park Y.B., Choi Y.S., Lee Y.W. (2000). Development of Korean activity scale/index (KASI). Korean Circ. J..

[28] Shaw L.J., Olson M.B., Kip K., Kelsey S.F., Johnson B.D., Mark D.B., Reis S.E., Mankad S., Rogers W.J., Pohost G.M. (2006). The value of estimated functional capacity in estimating outcome: Results from the NHBLI-Sponsored Women’s Ischemia Syndrome Evaluation (WISE) Study. J. Am. Coll. Cardiol..

[29] Rabin R., Charro F.d. (2001). EQ-SD: A measure of health status from the EuroQol Group. Ann. Med..

[30] Kim M.-H., Cho Y.-S., Uhm W.-S., Kim S., Bae S.-C. (2005). Cross-cultural adaptation and validation of the Korean version of the EQ-5D in patients with rheumatic diseases. Qual. Life Res..

[31] Lee Y.K., Nam H., Chuang L.H., Kim K.Y., Yang H.K., Kwon I.S., Kind P., Kweon S.S., Kim Y.T. (2009). South Korean time trade-off values for EQ-5D health states: Modeling with observed values for 101 health states. Value Health.

[32] Ware J.E. (2000). SF-36 health survey update. Spine.

[33] Han C.W., Lee E.J., Iwaya T., Kataoka H., Kohzuki M. (2004). Development of the Korean version of short-form 36-item health survey: Health related QOL of healthy elderly people and elderly patients in Korea. Tohoku J. Exp. Med..

[34] Hamm L.F., Crow R.S., Stull G.A., Hannan P. (1989). Safety and characteristics of exercise testing early after acute myocardial infarction. Am. J. Cardiol..

[35] Miller D.H., Borer J.S. (1982). Exercise testing early after myocardial infarction. Risks and benefits. Am. J. Med..

[36] Kim C., Park Y.B., Kim D.Y., Kim Y.J. (2010). The Safety of Early Exercise Stress Test after Coronary Intervention. J. Korean Acad. Rehabil. Med..

[37] Butman S.M., Olson H.G., Gardin J.M., Piters K.M., Hullett M., Butman L.K. (1984). Submaximal exercise testing after stabilization of unstable angina pectoris. J. Am. Coll. Cardiol..

[38] Haglund E.K., Bremander A.B. (2009). Aerobic capacity in patients with rheumatoid arthritis: A comparison of two submaximal test methods. Musculoskelet. Care.

[39] Stringer W.W. (2010). Cardiopulmonary exercise testing: Current applications. Expert Rev. Respir. Med..

[40] Takeyama J., Itoh H., Kato M., Koike A., Aoki K., Fu L.T., Watanabe H., Nagayama M., Katagiri T. (2000). Effects of physical training on the recovery of the autonomic nervous activity during exercise after coronary artery bypass grafting: Effects of physical training after CABG. Jpn. Circ. J..

[41] Wehrle A., Waibel S., Gollhofer A., Roecker K. (2021). Power Output and Efficiency During Supine, Recumbent, and Upright Cycle Ergometry. Front. Sports Act. Living.

[42] Pauletti H.O., Gomes W.J., Rocco I.S., Viceconte M., Garcia B.C.M., Marcondi N.O., Bublitz C.B., Costa A.D.S., Paiva T.P., Spina G.D. (2023). Early Six-Minute Walk Test May Predict Midterm Outcomes Following Coronary Artery Bypass Grafting. Braz. J. Cardiovasc. Surg..

[43] Doyle M.P., Indraratna P., Tardo D.T., Peeceeyen S.C., Peoples G.E. (2019). Safety and efficacy of aerobic exercise commenced early after cardiac surgery: A systematic review and meta-analysis. Eur. J. Prev. Cardiol..

[44] Alison J.A., Kenny P., King M.T., McKinley S., Aitken L.M., Leslie G.D., Elliott D. (2012). Repeatability of the six-minute walk test and relation to physical function in survivors of a critical illness. Phys. Ther..

